# Effective/cost effective interventions of child mental health problems in low- and middle-income countries (LAMIC): A protocol of systematic review

**DOI:** 10.1097/MD.0000000000018611

**Published:** 2020-01-03

**Authors:** Antonio Jose Grande, Wagner Silva Ribeiro, Christine Faustino, Claudio Torres de Miranda, David Mcdaid, Andra Fry, Silvia Helena Mendonça de Moraes, Sandra Maria do Valle Leone de Oliveira, Joni Marcio de Farias, Paulo de Tarso Coelho Jardim, Derek King, Valter Silva, Carolina Ziebold, Sara Evans-Lacko

**Affiliations:** a Universidade Estadual de Mato Grosso do Sul, Campo Grande, Brazil; b Care Policy and Evaluation Centre, Department of Health Policy, London School of Economics and Political Science, London, United Kingdom; c Departamento de Psiquiatria, Universidade Federal de São Paulo, São Paulo; d Universidade Federal de Alagoas, Maceió, Brazil; e Library, London School of Economics and Political Science, London, United Kingdom; f FIOCRUZ Mato Grosso do Sul, Campo Grande; g Universidade Federal de Mato Grosso do Sul, Campo Grande; h Centro Universitário Tiradentes, Maceió; i Universidade do Extremo Sul Catarinense, Brazil.

**Keywords:** Brazil, low- and middle-income countries, mental health services, neurodevelopmental disorders

## Abstract

**Background:**

This systematic review protocol aims to examine the evidence of effectiveness and cost-effectiveness of interventions for children and adolescents with, or at risk of developing mental disorders in low- and middle-income countries (LAMICs).

**Methods:**

We will search Medline Ovid, EMBASE Ovid, PsycINFO Ovid, CINAHL, LILACS, BDENF and IBECS. We will include randomised and non-randomised controlled trials, economic modelling studies and economic evaluations. Participants are 6 to 18 year-old children and adolescents who live in a LAMIC and who present with, or are at high risk of developing, one or more of the conditions: depression, anxiety, behavioural disorders, eating disorders, psychosis, substance abuse, autism and intellectual disabilities as defined by the DSM-V. Interventions which address suicide, self-harm will also be included, if identified during the extraction process. We will include in person or e-health interventions which have some evidence of effectiveness (in relation to clinical and/or functional outcomes) and which have been delivered to young people in LAMICs. We will consider a wide range of delivery channels (e.g., in person, web-based or virtual, phone), different practitioners (healthcare practitioners, teachers, lay health care providers) and sectors (i.e., primary, secondary and tertiary health care, education, guardianship councils). In the pilot of screening procedures, 5% of all references will be screened by two reviewers. Divergences will be resolved by one expert in mental health research. Reviewers will be retrained afterwards to ensure reliability. The remaining 95% will be screened by one reviewer. Covidence web-based tool will be used to perform screening of references and full text paper, and data extraction.

**Results:**

The protocol of this systematic review will be disseminated in a peer-reviewed journal and presented at relevant conferences. The results will be presented descriptively and, if possible, meta-analysis will be conducted. Ethical approval is not needed for anonymised secondary data.

**Conclusion:**

the systematic review could help health specialists and other professionals to identify evidence-based strategies to deal with child and adolescents with mental health conditions.

## Abstract 2

Supplemental Digital Content is available in the text

## 1 Introduction

### 1.1 Description of the conditions

In recent decades, the patterns of physical and mental illness in children and adolescents have changed considerably.^[1,2]^ Emotional and behavioral problems worldwide represent a significant disease burden and can lead to impairment in school life, family, and social relationships of affected children and adolescents.^[1]^ Worldwide mental illness is experienced by 10% to 20% of children and adolescents.^[2]^ About 50% of all mental illnesses have been estimated to begin around 14 years .^[3]^

In addition, mental health problems can persist over the lifetime, with a significant proportion of young people with mental illness having some impairment in adult life.^[4,5]^ The global burden of mental illness surpasses both cardiovascular disease and cancer in disability-adjusted life years (DALYs).^[6]^ Research on the global burden of disease in young people between 10 and 24 years showed that,^[7]^ worldwide, the three main causes of years lost because of disability in this age group are, respectively: neuropsychiatric disorders (45.0%), unintentional injuries (12.0%) and infectious and parasitic diseases (10.0%). Most prevalent neuropsychiatric disorders in childhood and adolescence include depression, anxiety and behavioral disorders,^[1]^ as well as substance-related disorders.^[8]^ Although less prevalent, eating disorders,^[9]^ autism spectrum disorders^[10]^ and psychotic disorders^[11]^ are associated with high levels of disability and impairment, and with reduced life expectancy.

In recent decades, there has been a good progress in developing and evaluating interventions to treat and prevent child and adolescent mental health problems in HIC, and there is now robust evidence of effective interventions for most child and adolescent psychiatric problems.^[12–16]^ However, resources and research are scarce in low- and middle-income countries (LAMICs)^[17]^ and the gap between needs and actual provision is greater for child and adolescent mental health care compared with adults.^[17–19]^ Children and adolescents constitute almost a third (2,2 billion individuals) of the world's population. Almost 90.0% of them live in LAMICs, where they form up to 50.0% of the population.^[12,20]^

### 1.2 Why it is important to do this review

Health professionals working in primary care settings, where most people with mental health problems could be treated, often do not have access to adequate training and/or technical resources to deliver prevention or treatment strategies to young people with mental health problems.^[21–23]^ To address this issue worldwide, the World Health Organization published the “Mental health action plan 2013–2020” in which children and adolescent mental health disorders emerge as requiring particular attention for prevention and care.^[2]^

### 1.3 Objectives

The aims of this systematic review are:

(1) to identify all evidence-based interventions for treatment and prevention (among high risk groups) for a set of child and adolescent mental health conditions which have been delivered and evaluated in LAMICs;
(2) to assess the effectiveness and cost-effectiveness of identified interventions;
(3) to compare interventions based on their nature, by who and where they are delivered and evidence of cross-sectoral collaboration (i.e., between the health and education system and guardianship councils).

## 2 Methods and analysis

The present systematic review will be conducted following this protocol. We reported this review based in Preferred Reporting Items for Systematic Reviews and Meta-Analyses (PRISMA).^[21]^

### 2.1 Inclusion criteria for study selection

#### 2.1.1 Types of studies

We will include any randomized or non-randomized controlled trials. We will also include economic modelling studies and economic evaluations.

#### 2.1.2 Types of participants

Participants are children and adolescents aged 6 to 18 years who live in Latin America or other LAMICs.

#### 2.1.3 Types of conditions

This review will include depressive, anxiety, behavioral and substance-related disorders, which are the most prevalent mental health problems among children and adolescents. It will also include eating, psychotic and autism spectrum disorders. Although less prevalent, these disorders are associated with high levels of disability and impairment, and with reduced life expectancy.

Suicide and self-harm behavior are not DSM-V diagnoses,^[24]^ but they are linked to many disorders. Although they were not included in our search strategy, we will include interventions to these conditions in our review if they are identified during data extraction.

#### 2.1.4 Study design

We will include experimental studies with the following study designs: randomized and non-randomized controlled trials. Economic evaluations linked to empirical studies, as well as economic modelling studies that synthesize data on costs and effects from multiple sources are also eligible for inclusion.

#### 2.1.5 Interventions

We will include in person or e-health interventions which have evidence of effectiveness (in relation to clinical and/or functional outcomes) and which have been delivered to young people in LAMICs. We will consider a wide range of delivery channels (eg, in person, online, phone), different practitioners (healthcare practitioners, teachers, lay health care providers) and sectors (ie, health, primary, secondary and tertiary care, education, guardianship councils).

#### 2.1.6 Primary outcomes

We will primarily include interventions designed to improve participants’ mental health status, either by reducing mental health symptoms or by improving mental health functioning.

#### 2.1.7 Secondary outcomes

Studies that did not assess our primary outcomes will still be included in the review if their interventions helped improve the following secondary outcomes related to mental health status: reduction in hospitalization; improvement of well-being, quality of life or functioning (physical, social, or occupational); economic impact.

#### 2.1.8 Language restrictions

No language restrictions will be used. In case of articles published in languages other than Portuguese, English and Spanish, we will use academic networks to translate the critical parts to enable screening of abstracts and, if necessary, critical parts of the full paper.

#### 2.1.9 Time restrictions

Only studies published from 2007 will be included, because this is the year in which child and adolescent mental health became prominent as a global public health challenge.^[25]^

### 2.2 Search methods for identification of studies

#### 2.2.1 Electronic searches

We will search MEDLINE Ovid (1946 to latest issue), EMBASE Ovid (1974 to latest issue), PsycINFO Ovid (1806 to latest issue), CINAHL plus (1937 to latest issue), LILACS (Latin American and Caribbean Health Sciences) (1982 to latest issue), BDENF (Brazilian Nursing Database) and IBECS (The Spanish Bibliographic Index of the Health Sciences). We will use the search strategy included in Appendix 1, to search MEDLINE and adapt it to all other databases. Search filters will be used to exclude comments, editorials and letters, as well as animal studies.

#### 2.2.2 Searching other resources

We will check the reference lists of all primary studies and review articles for additional references. We will email experts in the field about other published and unpublished studies that may be eligible for inclusion.

### 2.3 Data collection and analysis

#### 2.3.1 Pilot phase and screening of references

To ensure reliability between reviewers, we will run a pilot study in which 5% of all references will be independently screened by 2 different reviewers. An expert in mental health research (WSR) will resolve divergences and make the final decision. Based on the identification of the main reasons for divergences between reviewers, a meeting will be held with the review team to clarify potential doubts and solve any systematic error when screening references.

#### 2.3.2 Selection of studies

After divergences in the pilot phase are solved and the screening team is retrained, the remaining 95% of references will be equally split among the reviewers to finalize the screening phase. Based on our inclusion and exclusion criteria, reviewers will read titles and abstracts and will classify references into three categories: “no”, “yes”, and “maybe”. References classified as “no” will be excluded. Those classified as “yes” or “maybe” will be selected for the full text screening phase, and will be analyzed again against inclusion/exclusion criteria after full texts have been obtained and read.

The web-based Covidence (www.covidence.org) tool will be used to perform the management and screening of references, and data extraction from eligible studies.

### 2.4 Data extraction and management

A standardized, pre-piloted form will be used to extract data from the included studies, which will be used for the assessment of study quality and evidence synthesis. Missing data will be requested from study authors. The extraction sheet will include:

*Study details*: aim, study design, design details, country in which study was conducted, details on location of intervention delivery (ie, city or community), target condition or risk factor (ie, subthreshold symptoms, experience of child maltreatment).

*Participants:* sample size (intervention and control groups at baseline and follow-up), sociodemographic characteristics (eg, age, gender, ethnicity, socioeconomic status).

*Interventions:* description of intervention including frequency and duration, number of sessions, mode of delivery (eg, face to face, internet), format (eg, one to one or group), cost of intervention.

*Delivery of the intervention:* setting in which intervention was delivered (eg, school, home, healthcare practice), who delivered the intervention (eg, medical doctor, nurse, psychologist, teacher, lay health worker etc) and whether it was delivered by 1 practitioner or a team of individuals, whether there was intersectoral collaboration (eg, between health and education or guardianship councils).

*Comparison groups:* characteristics of and procedures for selection comparison groups (eg, matching vs randomization).

*Outcomes:* primary outcomes will include reduction of mental health symptoms, or improvement in mental health functioning. Secondary outcomes will include, economic impact, reduction of hospitalizations, or improvement in wellbeing, quality of life, resilience, social, physical and occupational functioning, including educational outcomes

A modified version of the pre-piloted form will be used for any economic evaluations that are identified. This will additionally include information on the design of the economic evaluation, perspective adopted, summary of information on resource use and costs and summary of cost-effectiveness results.

Two reviewers will extract data independently following literature recommendation.^[26]^ Data will be extracted using standardized forms and will be stored in RevMan 5.3 software.

### 2.5 Assessment of risk of bias in included studies

The methodological quality of the included studies will be independently evaluated by pairs in accordance with recommended guidelines.^[26]^

### 2.6 Measures of treatment effect

#### 2.6.1 Types of measurements of treatment effect that may be used

For dichotomous outcomes, we will use risk ratio (RR) to estimate treatment effects. When outcomes are presented as continuous data, we will combine results using the mean differences (MD) for measures using the same scale, or standardized mean differences (SMD) where different scales have been used to evaluate the same outcome.

### 2.7 Unit of analysis issues

We will consider the individual as the unit of analysis.

### 2.8 Dealing with missing data

We will send 2 emails (1 initial, 1 reminder) to corresponding authors to ask for any missing data or incompletely reported study details. We will check for consistency between studies and analyze each outcome.

### 2.9 Assessment of heterogeneity

We will assess heterogeneity between studies using the *I*^2^ statistic and describe the percentage of variability in effect. We will consider heterogeneity substantial if *I*^2^ is over 50%.

### 2.10 Assessment of reporting biases

If discrepancies are identified between study protocols and reports, we will contact the trial authors to clarify such discrepancies. We plan to explore the impact of including such studies by conducting a sensitivity analysis. We will perform a funnel plot asymmetry test if 10 or more trials are included.

### 2.11 Data synthesis

We will present results separately for randomized, non-randomized and economic evaluation studies. We will try to perform meta-analysis if results of different studies are combinable. We will use a random-effects model, regardless of heterogeneity between studies. Forest plot graphics produced by RevMan 5.3 will illustrate meta-analysis. If it is not possible to combine studies’ results, we will present a narrative analysis of individual studies. We will create a 'Summary of findings’ table using the outcomes proposed in this protocol and we will present the quality of the body of evidence considering the 5 assumptions of The Grading of Recommendations Assessment, Development and Evaluation (GRADE)^[27]^ – study limitations, consistency of effect, imprecision, indirectness and publication bias – contributing to the meta-analyses for the pre-specified outcomes.

### 2.12 Subgroup analysis and investigation of heterogeneity

We plan to explore the subsets or subgroups of countries and regions, care sector, type of mental health condition, gender, age, and area (urban or rural).

### 2.13 Sensitivity analysis

We will pool included studies to verify whether the impact of risk of bias affects the overall effect. We will explore which studies increase heterogeneity.

### 2.14 Grading the quality of evidence

Six quality criteria will be used, adapted from the Evidence for Policy and Practice Information and Coordinating Centre,^[28]^ assessing whether:

(1) aims are clearly stated,
(2) design is appropriate to the stated objectives,
(3) justification of sample size is reported,
(4) evidence of reliability or validity of measures used is reported,
(5) if statistical strategies are accurately reported,
(6) sample selection was relatively unbiased.

### 2.15 Ethics and dissemination

A formal ethical approval is not necessary because our study will deal with data that have been published in the scientific literature and, therefore, will not involve contact with research participants. The results will be disseminated through peer-reviewed publications or conference presentations.

## 3 Discussion

To the best of our knowledge, no systematic reviews related to interventions on mental health disorders for children and adolescents living in LMIC has been published. The evaluation of this systematic review will be divided into 4 sections: identification, study inclusion, data extraction, and data synthesis. We hope that this review will provide policymakers and practitioners with robust evidence to support the implementation of effective interventions to treat and prevent mental disorders among young people which are suitable to LMIC contexts, where resources are scarce. This review has some potential limitations. Different mental health conditions and criteria for efficacy evaluation may cause significant heterogeneity. Moreover, the limited time frame may result on potentially eligible studies being missed. However, it makes the search feasible, considering the number of mental health conditions we are including in our review.

## Author contributions

Antonio Jose Grande: conceived and designed the study; contributed to the definition of the search strategy; wrote the first version of the manuscript.

Wagner Silva Ribeiro: conceived and designed the study; contributed to the definition of the search strategy and the writing up of the manuscript.

Chrsitine Faustino: conceived and designed the study; contributed to the definition of the search strategy and revision of the manuscript.

Claudio Torres de Miranda: contributed to the study design and revision of the manuscript

David Mcdaid: contributed to the study design, definition of the search strategy and revision of the manuscript

Andra Fry: designed the search strategy; contributed to the study design and revision of the manuscript

Silvia Helena Mendonça de Moraes: conceived and designed the study; contributed to the definition of the search strategy and revision of the manuscript.

Sandra Oliveira: designed the search strategy; conceived and designed the study; contributed to the definition of the search strategy and revision of the manuscript.

Joni Marcio de Farias: conceived and designed the study; contributed to the definition of the search strategy and revision of the manuscript.

Paulo de Tarso Coelho Jardim: conceived and designed the study; contributed to the definition of the search strategy and revision of the manuscript.

Derek King: conceived and designed the study; contributed to the definition of the search strategy and revision of the manuscript.

Valter Silva: designed the search strategy; conceived and designed the study; contributed to the definition of the search strategy and revision of the manuscript.

Carolina Ziebold: contributed to the study design, definition of search strategy and revision of the manuscript

Sara Evans-Lacko: conceived and designed the study; contributed to the definition of the search strategy and revision of the manuscript.

Antonio Jose Grande orcid: 0000-0001-7182-075X.

## Supplementary Material

Supplemental Digital Content
