# Supplementary material for: Effective/cost effective interventions of child mental health problems in low- and middle-income countries (LAMIC): A protocol of systematic review
Source: Medicine (Baltimore). 2020 Jan 3;99(1):e18611. doi: 10.1097/MD.0000000000018611 (PMC6946409; doi:10.1097/MD.0000000000018611)
Supplement: Supplemental Digital Content [file medi-99-e18611-s001.docx]

Medline Ovid

1 Child/

2 child*.tw.

3 schoolchild*.tw.

4 pupil*.tw.

5 (kid or kids).tw.

6 (boy* or girl*).tw.

7 Minors/

8 minors.tw.

9 underage*.tw.

10 under-age*.tw.

11 juvenil*.tw.

12 Adolescent/

13 adolescen*.tw.

14 teen*.tw.

15 youth*.tw.

16 preadolescen*.tw.

17 Puberty/

18 pubert*.tw.

19 pubescen*.tw.

20 prepube*.tw.

21 pre-pube*.tw.

22 Pediatrics/

23 pediatric*.tw.

24 paediatric*.tw.

25 peadiatric*.tw.

26 Schools/

27 school*.tw.

28 1 or 2 or 3 or 4 or 5 or 6 or 7 or 8 or 9 or 10 or 11 or 12 or 13 or 14 or 15 or 16 or 17 or 18 or 19 or 20 or 21 or 22 or 23 or 24 or 25 or 26 or 27

29 Developing Countries.sh,kf.

30 (Africa or Asia or Caribbean or West Indies or South America or Latin America or Central America).hw,kf,ti,ab,cp.

31 (Afghan* or Albania* or Algeria* or Angola* or Anguilla* or Antigua* or Barbuda* or Argentin* or Armenia* or Azerbaijan* or Azeri or Bangladesh* or Barbad* or Benin* or Byelarus or Byelorussian or Belarus* or Belorussia* or Belize* or Bhutan* or Bolivia* or Bosnia* or Herzegovin* or Hercegovin* or Botswana or Botsuana or Motswana or Batswana or Brasil* or Brazil* or Burkina Faso or Burkina Fasso or Burkina* or Burundi* or Urundi* or Cambodia* or Cameroon* or Cameron* or Cape Verd* or Cabo Verde or Central African Republic or Chad* or Tchad* or Chile* or China or Chinese or Colombia* or Columbia* or Comoros or Comoro Islands or Comores or Comoran or Mayotte or Congo* or Costa Rica* or Cote d'Ivoire or Ivory Coast or Ivorian* or Cook Islands or Cuba* or Croat* or Djibouti* or Dominica* or East Timor or East Timur or Timor Leste or Timorese or Ecuador* or Equador* or Egypt* or El Salvador or Salvadoran or Eritrea* or Ethiopia* or Fiji* or Gabon* or Gambia* or Gaza or Georgia Republic or Georgian or Abkhazia* or Abchasia* or South Ossetia* or Ghana* or Grenada or Grenadian or Guatemala* or Guinea* or Guinea Bissau or Guian* or Guyana or Haiti* or Hondura* or India or Indian or Indonesia* or Iran* or Iraq* or Jamaica* or Jordan* or Kazakhstan* or Kazakh or Kenya* or Kiribati or Korea* or Kosovo or Kosova* or Kyrgyzstan or Kirghizia or Kyrgyz or Kirghiz or Kirgizstan or Lao PDR or Laos or Laotian or Lebanon or Lebanese or Lesotho or Mosotho or Basotho or Liberia* or Libya* or Macedonia* or FYROM or Madagasca* or Malagasy or Malaysia* or Malaya* or Malay or Sabah or Sarawak or Malawi* or Maldives or Maldivan or Mali or Malian or Marshall Islands or Marshallese or Mauritania* or Mauriti* or Agalega Islands or Mexico or Mexican or Micronesia* or Middle East* or Moldova* or Moldovia* or Transnistria* or Mongolia* or Montenegr* or Montserrat* or Morocc* or Mozambique or Mozambican or Myanmar* or Myanma or Burma or Burmese or Namibia* or Nauru* or Niue or Nepal* or Nicaragua* or Niger or Nigerien or Nigeria* or Oman* or Pakistan* or Palau* or Palestine or Palestinian or Panama or Panamanian or Paraguay* or Papua New Guinea* or Peru or Peruvian or Philippines or Philipines or Phillipines or Phillippines or Filipino or Philipino or Philippino or Phillipino or Phillippino or Rwanda* or Ruanda* or Saint Helen* or St Helen* or Saint Kitts or St Kitts or Kittian or Nevis* or Saint Lucia* or St Lucia* or Saint Vincent or St Vincent or Vicentian or Grenadines or Samoa* or Sao Tome* or Senegal* or Serbia* or Seychell* or Sierra Leone* or Sri Lanka* or Ceylon or Solomon Island* or Somali* or South Africa* or Sudan or Sudanese or Surinam* or Swaziland or Swazi or Eswatini or Syria or Syrian or Tajikistan or Tadzhikistan or Tadjikistan or Tajik or Tadzhik or Tanzania* or Thailand or Thai or Togo or Togolese or Tonga* or Tunisia* or Tokelau or Trinidad* or Tobago* or Turkey or Turkish or Turks or Turkmenistan or Turkmen or Tuvalu* or Uganda* or Ukraine or Ukrainian or Uruguay* or Uzbekistan* or Uzbek or Vanuatu or Venezuela* or Vietnam* or Viet Nam or Wallis Futuna or West Bank or Yemen* or Zambia* or Zimbabwe*).hw,kf,ti,ab,cp.

32 ((developing or less* developed or under developed or underdeveloped or middle income or low* income or underserved or under served or deprived or poor*) adj (countr* or nation? or population? or world)).ti,ab.

33 ((developing or less* developed or under developed or underdeveloped or middle income or low* income) adj (economy or economies)).ti,ab.

34 (low* adj (gdp or gnp or gross domestic or gross national)).ti,ab.

35 (low adj3 middle adj3 countr*).ti,ab.

36 (lmic or lmics or third world or lami countr*).ti,ab.

37 transitional countr*.ti,ab.

38 29 or 30 or 31 or 32 or 33 or 34 or 35 or 36 or 37

39 randomized controlled trial.pt.

40 controlled clinical trial.pt.

41 (randomized or randomised).ab.

42 placebo.ab.

43 drug therapy.fs.

44 randomly.ab.

45 trial.ab.

46 groups.ab.

47 39 or 40 or 41 or 42 or 43 or 44 or 45 or 46

48 randomized controlled trial.pt.

49 (cluster* adj2 randomi*).tw.

50 ((communit* adj2 intervention*) or (communit* adj2 randomi*)).tw.

51 group* randomi*.tw.

52 intervention?.tw.

53 cluster analysis/

54 48 or 49 or 50 or 51 or 52 or 53

55 health promotion/

56 program evaluation/

57 health education/

58 (health adj2 (educat* or program* or promot*)).tw.

59 program* evaluation*.tw.

60 55 or 56 or 57 or 58 or 59

61 risk*.tw.

62 exp cohort studies/

63 between group*.tw.

64 (non random* or nonrandom*).tw.

65 Non-Randomized Controlled Trials as Topic/

66 quasi*.tw.

67 (natural adj3 experiment*).tw.

68 instrumental variable*.tw.

69 cohort*.tw.

70 (before adj2 after).tw.

71 Controlled Before-After Studies/

72 (difference-in-difference* or diff-in-diff*).tw.

73 regression discontinuity.tw.

74 Historically Controlled Study/

75 historical* control*.tw.

76 Interrupted Time Series Analysis/

77 interrupted Time Series.tw.

78 (case* and control*).tw.

79 Case-Control Studies/

80 match*.tw.

81 propensity.tw.

82 Propensity Score/

83 61 or 62 or 63 or 64 or 65 or 66 or 67 or 68 or 69 or 70 or 71 or 72 or 73 or 74 or 75 or 76 or 77 or 78 or 79 or 80 or 81 or 82

84 economics/

85 exp "costs and cost analysis"/

86 exp economics, hospital/

87 exp economics, medical/

88 economics, nursing/

89 economics, pharmaceutical/

90 budgets/

91 exp models, economic/

92 markov chains/

93 monte carlo method/

94 decision trees/

95 econom*.tw.

96 (cba or cea or cua).tw.

97 markov*.tw.

98 (monte adj carlo).tw.

99 (decision* adj3 (tree* or analy* or model*)).tw.

100 (cost or costs or costing* or costly or costed).tw.

101 (price* or pricing*).tw.

102 budget*.tw.

103 expenditure*.tw.

104 (value adj3 (money or monetary)).tw.

105 (pharmacoeconomic* or (pharmaco adj economic*)).tw.

106 84 or 85 or 86 or 87 or 88 or 89 or 90 or 91 or 92 or 93 or 94 or 95 or 96 or 97 or 98 or 99 or 100 or 101 or 102 or 103 or 104 or 105

107 47 or 54 or 60 or 83 or 106

108 Mental Health/

109 Mental Disorders/

110 Mentally Ill Persons/

111 ((mental* or psychiatric*) adj2 (health or ill or illness* or disorder* or disease* or problem* or wellbeing or well-being or wellness)).tw.

112 CAMH.tw.

113 108 or 109 or 110 or 111 or 112

114 Depressive Disorder/

115 Depressive Disorder, Major/

116 Depressive Disorder, Treatment-Resistant/

117 Depression/

118 (depression* or depressive* or depressed).tw.

119 114 or 115 or 116 or 117 or 118

120 respiratory depression.tw.

121 119 not 120

122 Anxiety Disorders/

123 Anxiety/

124 Phobia, Social/

125 Anxiety, Separation/

126 (anxiet* or anxious*).tw.

127 122 or 123 or 124 or 125 or 126

128 Adjustment Disorders/

129 Problem Behavior/

130 Child Behavior Disorders/

131 "Attention Deficit and Disruptive Behavior Disorders"/

132 Attention Deficit Disorder with Hyperactivity/

133 Conduct Disorder/

134 (behavio* adj5 (disorder* or disturb* or problem* or difficult*)).tw.

135 (conduct adj5 (disorder* or disturb* or problem* or difficult)).tw.

136 (adjustment adj5 (disorder* or disturb* or problem* or difficult*)).tw.

137 oppositional*.tw.

138 ((antisocial* or anti-social*) adj5 (behav* or histor* or conduct)).tw.

139 (attention adj5 deficit*).tw.

140 (adhd or "ad/hd" or adhkd or addh or adhs).tw.

141 hyperactiv*.tw.

142 128 or 129 or 130 or 131 or 132 or 133 or 134 or 135 or 136 or 137 or 138 or 139 or 140 or 141

143 anorexia nervosa/ or binge-eating disorder/ or bulimia nervosa/

144 Bulimia/

145 Anorexia/

146 (eat* adj5 disorder*).tw.

147 anorexi*.tw.

148 bulimi*.tw.

149 (binge* adj5 eat*).tw.

150 EDNOS.tw.

151 143 or 144 or 145 or 146 or 147 or 148 or 149 or 150

152 Psychotic Disorders/

153 psychoses, substance-induced/ or psychoses, alcoholic/

154 Affective Disorders, Psychotic/

155 (psychotic* or psychosis or psychoses).tw.

156 152 or 153 or 154 or 155

157 Substance-Related Disorders/

158 alcohol-related disorders/ or alcohol-induced disorders/

159 ((alcohol* or drug* or substance*) adj3 (abus* or dependen* or addict* or misus* or disorder*)).tw.

160 157 or 158 or 159

161 Suicide/

162 Suicidal Ideation/

163 Suicide, Attempted/

164 suicid*.tw.

165 parasuicid*.tw.

166 161 or 162 or 163 or 164 or 165

167 Autism Spectrum Disorder/

168 asperger syndrome/ or autistic disorder/

169 Child Development Disorders, Pervasive/

170 (autistic* or autism*).tw.

171 asperger*.tw.

172 167 or 168 or 169 or 170 or 171

173 Intellectual Disability/

174 Mentally Disabled Persons/

175 (intellect* adj3 (defici* or difficult* or disab* or disorder* or handicap* or impair* or retard* or incapacit* or subaverage or sub-average or subnorm* or sub-norm* or dysfunction or defect*)).tw.

176 (mental* adj2 (disab* or disorder* or handicap* or impair* or defici* or subnorm* or sub-norm* or retard*)).tw.

177 (low*2 adj2 intellect*).tw.

178 (mental* adj3 retard*).tw.

179 173 or 174 or 175 or 176 or 177 or 178

180 113 or 121 or 127 or 142 or 151 or 156 or 160 or 166 or 172 or 179

181 28 and 38 and 107 and 180

182 exp animals/ not humans/

183 181 not 182

184 (comment or editorial or letter).pt.

185 183 not 184

186 limit 185 to yr="2007 -Current"
